# Doxorubicin-Loaded Mixed Micelles Using Degradable Graft and Diblock Copolymers to Enhance Anticancer Sensitivity

**DOI:** 10.3390/cancers13153816

**Published:** 2021-07-29

**Authors:** Yi-Chun Chen, Chang-Jung Chang, Ging-Ho Hsiue, Yi-Ting Chiang

**Affiliations:** 1 Department of Forestry, National Chung Hsing University, Taichung City 402, Taiwan; chenyc@nchu.edu.tw; 2 Department of Chemical Engineering, National Tsing Hua University, Hsinchu 300, Taiwan; kymco741120@hotmail.com; 3 Department of Chemical Engineering, National Chung Hsing University, Taichung City 402, Taiwan; 4 School of Pharmacy, China Medical University, Taichung City 406, Taiwan

**Keywords:** copolymer, doxorubicin, drug release, micelle, pH responsive

## Abstract

**Simple Summary:**

In this study, a long-circulating and pH responsive mixed micellar system was assembled with a degradable graft copolymer, poly(*N*-(2-hydroxypropyl) methacrylamide dilactate)-*co*-(*N*-(2-hydroxypropyl) methacrylamide-*co*-histidine)-graft-poly(d,l-lactide), and a diblock copolymer, methoxy poly(ethylene glycol)-*b*-poly(d,l-lactide) to load with the anticancer agent doxorubicin. The in vitro results indicate that the micellar system display high biosafety and intracellular drug-releasing behavior in cancer cells. Furthermore, the in vivo results show that the high stability of the mixed micelles leads to a high tumor accumulation and hence an excellent inhibition of tumor growth. This mixed micellar system, comprising degradable diblock and graft copolymers enables one to increase cancer cells’ sensitivity toward doxorubicin (Dox) and is feasible for further clinical use in cancer therapy.

**Abstract:**

In this study, a graft copolymer, poly(*N*-(2-hydroxypropyl) methacrylamide dilactate)-*co*-(*N*-(2-hydroxypropyl) methacrylamide-*co*-histidine)-graft-poly(d,l-lactide), and a diblock copolymer, methoxy poly(ethylene glycol)-*b*-poly(d,l-lactide), were assembled into a mixed micellar system to encapsulate the anticancer drug doxorubicin (Dox). This mixed micellar system possesses the hydrophobic lactide segment of both copolymers, which reinforces its stability in physiological milieus; the histidine molecules appended on the graft copolymer provide the desired pH-responsive behavior to release Dox during internalization in cancer cells. The results demonstrate that the two copolymers were successfully prepared, and their ratios in the mixed micelles were optimized on the basis of the results of the stability tests. Under acidic conditions, the mixed micelles swell and are able to release their payloads. Therefore, the in vitro results indicate that the Dox in the mixed micelles is released effectively in response to the environmental pH of the mimetic internalization process, increasing cancer cells’ sensitivity toward Dox. The mixed micelles display low cytotoxicity due to the degradability of the polymers. The in vivo images show that the high stability of the mixed micelles ensures a high tumor accumulation. This selective tumor accumulation results in an excellent inhibition of in vivo tumor growth and a high rate of apoptosis in cancerous tissues, with low toxicity. This highly stable, mixed micellar system with a pH-dependent drug release, which enables the precise delivery of drugs to the tumor lesions, is feasible to employ clinically in cancer therapy.

## 1. Introduction

Polymeric mixed micelles comprise two or more polymers and spontaneously assemble into a core-shell nanostructure as they achieve the critical micellar concentration (CMC). In aqueous milieus above the CMC, the hydrophobic segments of the polymers form the core inside the micelles, and the hydrophilic parts distribute outside as a shell layer around the micellar structures [1]. The core of the mixed micelles therefore offers a space in which to load hydrophobic moieties, and their water solubility can be enhanced via encapsulation into the mixed micelles. Polymeric mixed micelles have been employed as a drug delivery system to improve the solubility of potent hydrophobic reagents [2,3]. In recent years, long-circulating polymeric mixed micelles, which have low CMC values, have been extensively investigated for their selective and tumor-specific accumulation via the angiogenesis vessels of oncological lesions [4,5]. The enhancement in the sensitivity of the loading of anticancer reagents on long-circulating micelles has been reported [6]. Many polymeric mixed micelles have been designed to carry hydrophobic antineoplastic reagents to treat cancers.

To precisely release the payloads of polymeric mixed micelles in tumor tissues or cells, stimuli-sensitive mixed micelles were prepared based on the tumor tissues or intracellular environments [2]. The pH-responsive polymeric mixed micelles have been extensively studied, since the tumor tissue microenvironment is mildly acidic and the intracellular milieus becomes more acidified during endocytosis [7,8,9]. To fabricate a pH-responsive polymeric mixed micelle, functional polymers which enable the response to the environment, pH was introduced to the micellar system [2,3]. For example, Torchilin et al. and Bae et al. introduced the pH-responsive poly(ethylene glycol)-*b*-poly(histidine) copolymer into a polymeric mixed micellar system. In this copolymer, the imidazole ring of the histidine displays neutral charge in physiological conditions (pH 7.4), whereas displays encounters protonation and positive charge when the pH is lower than 6.5. Therefore, with decreasing pH, micellar structures containing poly(histidine) are gradually destroyed via electrostatic repulsion [10,11,12].

**Scheme 1:**
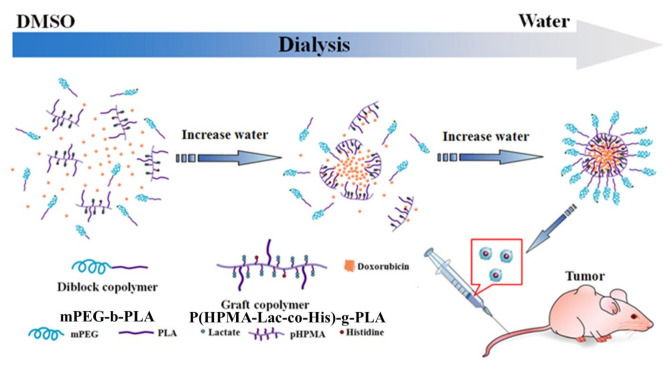
Schematic representation of the mixed micelle system comprising the graft copolymer P(HPMA-Lac-*co*-His)-*g*-PLA and diblock copolymer mPEG-*b*-PLA. The two polymers were assembled into mixed micelles via dialysis methods. The anticancer reagent, doxorubicin, was encapsulated into the mixed micellar system and the in vivo anticancer therapeutics were estimated.

In this study, we established a new pH-responsive mixed micelles system from a histidine-containing graft copolymer, poly(*N*-(2-hydroxypropyl) methacrylamide dilactate)-*co*-(*N*-(2-hydroxypropyl) methacrylamide-*co*-histidine)-graft-poly(d,l-lactide) (P(HPMA-Lac-*co*-His)-*g*-PLA), and diblock amphiphilic copolymer methoxy poly(ethylene glycol)-*b*-poly(d,l-lactide) (mPEG-*b*-PLA), as shown in Scheme 1. The histidine moieties were conjugated onto the hydrophilic *N*-(2-hydroxypropyl) methacrylamide polymer, which is known to be highly hydrophilic, biocompatible, long-circulating in vivo, and is applied as a polymer–drug conjugate at the clinical level [13,14]. Hennink et al. synthesized copolymers of HPMA-lactate via hydrophobizing by esterification with lactic acid to reduce the CMC value for application in a micellar system [15,16,17,18]. Additionally, the diblock amphiphilic copolymer mPEG-*b*-PLA was approved by the FDA as a micellar formulation to enhance the water solubility of hydrophobic anticancer reagents [19]. In this study, the hydrophobic lactate segments of the two polymers and the HPMA-dilactate of the graft copolymer form the core to encapsulate the hydrophobic anticancer reagent Dox, and the PEG of the diblock copolymer acts as a hydrophilic shell to stabilize the system and prevent the recognition of the micelles by monocytes [20,21]. The graft copolymers with hydrophobic segments enable to efficiently reduce the CMC value and enhance the stability of a mixed micellar system, as previously reported, [22,23]. Under acidic conditions, such as those found in tumor tissues or within tumor cells, the histidine molecules appended on the graft copolymers are protonated, collapsing the micellar structures and releasing Dox [24]. Notably, mPEG-*b*-PLA and HPMA-dilactate in the graft copolymer enable the hydrolysis of lactate groups [15,16,17,18,25,26]; hence, our mixed micellar system is biodegradable after releasing the drug and have the high biosafety, offering potential for further clinical application. In this study, the two polymers were prepared and characterized, and their ratios in the mixed micellar system were optimized. The physicochemical properties, morphology, stability and pH-responsiveness of the mixed micelles were investigated; the drug loading, release profiles, and the in vitro cytotoxicity of the Dox-mixed micelle were determined. Finally, the tumor targeting and anti-tumor therapeutic efficacy of the mixed micelles were assessed in a cancer xenograft model.

## 2. Materials and Methods

### 2.1. Materials and Animals

The polymers, including α-*t*-butyloxy-carbonylamino-ω-hydroxy-poly(ethylene glycol) (Boc-NH-PEG-OH) (molecular weight: 3000) as well as methoxy poly(ethylene glycol) (mPEG) (molecular weight: 5000) were obtained from Iris Biotech GmbH (Marktredwitz, Germany). d,l-Lactide, palladium, and the organic solvent tetrahydrofuran (THF), were purchased from Alfa Aesar (Ward Hill, MA, USA). The fluorescent dye, Cy5.5 NHS ester, was acquired from GE Healthcare (Chicago, IL, USA). The *N*-(2-hydroxypropyl) methacrylamide (HPMA) monomer was obtained from Polysciences, Inc. (Warrington, PA, USA). Other chemical reagents, including triethylamine (TEA), dimethyl sulfoxide (DMSO), sodium hydroxide (NaOH), the catalyst stannous octoate (Sn(Oct)_2_), and the free radical initiator 4,4′-azobis(4-cyanovaleric acid) (ABCPA) were all purchased from Millipore Sigma (Burlington, VT, USA). The reagents for the cell culture, including bovine serum albumin (BSA) and the salts for phosphate buffering saline (PBS) preparation, including potassium hydrogen phosphate, sodium chloride, potassium chloride, and disodium hydrogen phosphate, were also all obtained from Millipore Sigma. Chemical reagents, including *N*-(*tert*-butoxycarbonyl)-l-histidine (Boc-His), 4-(dimethylamino)pyridine (DMAP) and 1-(3-dimethylaminopropyl)-3-ethylcarbodiimide (EDC) were purchased from Tokyo Chemical Industry Co., Ltd. (Chuo-ku, Tokyo, Japan). The organic solvents, including diethyl ether, dichloromethane (DCM), and ethanol (EtOH) were all acquired from Tedia Company, Inc. (Fairfield, OH, USA). The fluorescent dyes, including LysoSensor™ Blue DND-167 and 4′6-diamidino-2-phenylindole dihydrochloride (DAPI) were purchased from Invitrogen (Thermo Fisher Scientific, Inc., Waltham, MA, USA). Dulbecco’s modified Eagle’s medium (DMEM) and penicillin–streptomycin reagent for the cell cultures were obtained from Gibco (Thermo Fisher Scientific), and fetal bovine serum (FBS) was acquired from Invitrogen (Thermo Fisher Scientific). The ApopTag^®^ Fluorescein In Situ Apoptosis Detection Kit S7110 was purchased from Merck Millipore (Burlington, MA, USA). The human ovarian carcinoma ES2 (ES2) and mouse Lewis lung carcinoma (LL/2) cell lines were obtained from the Food Industry Research and Development Institute (Hsinchu, Taiwan). The animal tests were approved by the Institutional Animal Care and Use Committee (IACUC) of the National Tsing Hua University and the China Medical University (IACUC approval number: CMUIACUC-2018-154). The BALB/c nude mice for the animal tests were provided by National Laboratory Animal Center (Taipei City, Taiwan, China).

### 2.2. Synthesis and Characterization of mPEG-b-PLA

The mPEG-*b*-PLA diblock copolymer was synthesized by ring-opening polymerization [22,27,28]. mPEG (Mw = 5000) (1 mmol) and d,l-lactide (25 mmol) was placed in a two-necked round-bottle flask with a magnetic stirrer, and toluene was added for dissolution under stirring. Afterward, the catalyst stannous octoate (Sn(Oct)_2_) (1 wt %) was added dropwise to initiate the ring-opening polymerization at 130 °C for 16 h under nitrogen. Finally, the reaction was terminated by adding 0.1 N methanolic KOH, and the product was obtained via precipitation from iced diethyl ether. The product was dried in a vacuum oven. The chemical structure of mPEG-*b*-PLA was characterized using a proton nuclear magnetic resonance (^1^H-NMR) spectrometer (Varian INOVA500 MHz spectrometer, Varian Inc., Palo Alto, CA, USA).

### 2.3. Synthesis and Characterization of P(HPMA-Lac-co-His)-g-PLA

To fabricate the graft copolymer, P(HPMA-Lac-*co*-His)-*g*-PLA, the PLA with a methacrylated group end-cap (HPMA-PLA, molecular weight = 1630) was prepared first. The HPMA-PLA moieties were further reacted with AIBN, HPMA, HPMA-dilactate, and HPMA-Boc-His to form the P(HPMA-Lac-*co*-Boc His)-*g*-PLA. After removing the protective group, Boc, via the hydrogen reaction, the P(HPMA-Lac-*co*-His)-*g*-PLA graft copolymer was obtained, as shown in Scheme S1 in the Supporting Information.

Firstly, the HPMA-PLA with a methacrylated group end-cap was synthesized via ring-opening polymerization [22]. In brief, HPMA (1 mmol) and d,l-lactide (3.6 or 7 mmol) were dissolved into dry toluene in a two-necked round-bottle flask under stirring. The catalyst, Sn(Oct)_2_, was gradually dropped into the two-necked round-bottle flask to initiate the polymerization at 130 °C under nitrogen [15,26]. After 16 h of polymerization, 0.1 N methanolic KOH was added to terminate the reaction, and the product was precipitated from diethyl ether at 0 °C. The product was dried in a vacuum oven for 24 h and its chemical structure was characterized using ^1^H-NMR after being re-dissolved in CDCl_3_.

The required HPMA-PLA moieties, which comprised different PLA units, were further reacted with AIBN, HPMA, HPMA-dilactate, and HPMA-Boc-His to acquire P(HPMA-Lac-*co*-His)-*g*-PLA graft copolymers. In advance, the HPMA-dilactate monomer was prepared. The HPMA-dilactate was synthesized via a ring-opening reaction. HPMA (1 mmol), d,l-lactide (2 mmol) was dissolved into dry toluene in a two-necked round-bottle flask under stirring. The catalyst, Sn(Oct)_2_, was gradually dropped into the two-necked round-bottle flask to initiate the reaction at 110 °C under nitrogen. After 5 h reaction, the methanolic KOH (0.1 N) was dropwise added into the solution in iced bath. The product was purified with silica gels and dried in a vacuum oven for 24 h. The chemical structures were determined by ^1^H-NMR. Afterwards, the graft copolymers were synthesized via free radical copolymerization. Briefly, the HPMA-containing molecules, including HPMA, HPMA-dilactate [15], HPMA-PLA, and HPMA-Boc-His [16], were all dissolved together with an initiator, AIBN, in DMSO in a two-necked round-bottle flask under nitrogen. The reaction was conducted at 70 °C in an oil bath for 24 h under nitrogen. Afterward, the product was purified by precipitation from iced diethyl ether. The copolymer, which was dried in a vacuum oven, was further dissolved in ethanol with a Pd catalyst and reacted with hydrogen at room temperature for 24 h. Thereafter, the Pd catalysts were removed using filtration, and the products were dried in a vacuum oven for 24 h. The dried polymer was re-dissolved in DMSO-d_6_ and characterized using its ^1^H-NMR spectrum.

The degradation of the graft copolymer P(HPMA-Lac-*co*-His)-*g*-PLA was determined using gel permeation chromatography (Shimadzu Corporation, Kyoto, Japan). The P(HPMA-Lac-*co*-His)-*g*-PLA copolymer was dispensed in pH 7.4 phosphate-buffered saline (PBS; 25 mg/mL). After reacted at 37 at 37 °C for 24, 48, 72, and 96 h post-incubation, the polymer solutions were freeze-dried and re-dissolved into THF. After filtration, the polymer solutions were analyzed using a GPC system with a 1 mL/min flow rate.

### 2.4. Preparation and Characterization of Polymeric Mixed Micelles

Mixed micelles were prepared using dialysis, following our previous method [23,24,29]. In brief, the graft copolymer P(HPMA-Lac-*co*-His)-*g*-PLA and diblock copolymer mPEG-*b*-PLA, whose total weight was 30 mg, were first dissolved together in 10 mL DMSO. The polymer solutions were placed in dialysis bags and dialyzed against the deionized water at 25 °C for 72 h. Afterward, the particle size and size distribution of the polymeric mixed micelles were directly determined using dynamic light scattering (DLS; Zetasizer 3000 HS, Malvern Panalytical, Worcestershire, UK). The correlation functions from DLS were analyzed using the constrained regularized CONTIN method. Additionally, the CMC value for the block copolymers was determined using fluorescent pyrene probe and analyzed from a plot of the intensity ratio: I_337.5_/I_335.5_ [30].

### 2.5. Drug Loading and Characterization

The Dox-loaded polymeric mixed micelles were prepared following the abovementioned procedures. Firstly, 10 mg of P(HPMA-Lac-*co*-His)-*g*-PLA graft copolymer and 20 mg mPEG-*b*-PLA diblock copolymer were dissolved in 5 mL DMSO to prepare a polymer solution. Afterward, various weight ratios of Dox hydrochloride solutions (5 mL) with TEA in a molar ratio of 1.2:1 were separately added to the polymer solution. After homogeneous blending, the mixed solution was dialyzed against water at 25 °C for 72 h and the distilled water was replaced per 3 h. When the dialysis was completed, the Dox-loaded polymeric mixed micelles were collected and freeze-dried to yield dried Dox-loaded micellar powder. The dried powder was weighed and re-dissolved in DMSO, and the Dox contents were measured using a UV–Vis spectrometer at 485 nm, referencing a calibration curve for Dox in DMSO to determine the drug contents and encapsulation efficiency (EE) of the Dox-mixed micelles. The drug content and encapsulation efficiency were calculated using the following formulas:

Drug content (%w/w) = (weight of Dox)/(weight of Dox-mixed micelle) × 100%;
Encapsulation efficiency (%w/w) = (weight of Dox)/(weight of infeed Dox) × 100%.

### 2.6. Drug Release Profiles of Dox-Mixed Micelles

The Dox-mixed micelle solution (50 mg/L) was placed in a cellulose membrane dialysis bag (molecular weight cutoff = 6000–8000) and the dialysis bag was placed at 37 °C in different pH environments (pH 7.4, 6.6, 5.4, and 4.5). At pre-determined time points, the released Dox was collected and freeze-dried. The dried Dox was dissolved in DMSO and its UV absorption was measured at a 485 nm wavelength using UV–Vis spectrometry.

### 2.7. Cytotoxicity Assessment

The cytotoxicity of blank mixed micelles, small-molecule Dox, and Dox-mixed micelles toward human ovarian carcinoma ES2 (ES2) and mouse Lewis lung carcinoma (LL/2) were determined using the MTT assay. In brief, the human ovarian carcinoma ES2 and mouse Lewis lung carcinoma (5 × 10^3^ cell) cells were first seeded on each well of a 96-well plate. The cells were cultured with DMEM, 10% FBS, and 1% penicillin/streptomycin, and incubated at 37 °C with a 5% CO_2_ supply for 12 h. When the cells were attached on the well, the cells were treated with various concentrations of blank mixed micelles, Dox, and Dox-mixed micelles; the concentrations of the Dox-mixed micelles were adjusted in advance based on the Dox concentration. After 24 and 72 h of co-culturing, the Dox and Dox-mixed micelles were removed and the cells were washed twice with PBS. The cell viability was determined using an MTT assay.

### 2.8. Observation of the Intracellular Drug-Releasing Behaviors in Cancer Cells and Internalization

A confocal laser scanning microscopic (CLSM) system (TCS SP5 Confocal Spectral Microscope Imaging System, Leica, Wetzlar, Germany) was utilized to witness the internalization and intracellular drug-releasing behaviors of the Dox and Dox-mixed micelles. In advance, the Dox-mixed micelles were labeled using a fluorescent dye, Cy 5.5, on the NH_2_-PEG-*b*-PLA diblock copolymer. The amine-capped diblock copolymer was first synthesized from Boc-NH-PEG (molecular weight = 3000) and d,l-lactide via ring-opening polymerization [22,24]. Briefly, d,l-lactide and Boc-NH-PEG were co- dissolved in toluene in a two-necked round-bottle flask. The catalyst, stannous octoate, was added dropwise under stirring to initiate the polymerization at 130 °C under nitrogen. After 16 h of reaction, 0.1 N of methanolic KOH was added to terminate the reaction. The product, Boc-NH-PEG-PLA, was obtained via precipitation from DCM and diethyl ether co-solvent. Boc-NH-PEG-PLA was further reacted with H_2_ in the presence of a Pd catalyst for 24 h to remove the Boc protective group. The final product, NH_2_-PEG-*b*-PLA diblock copolymer, was obtained after filtering out the Pd catalyst and precipitation from diethyl ether. The NH_2_-PEG-*b*-PLA diblock copolymer was dissolved in DMSO along with the fluorescent Cy5.5 NHS ester dye at room temperature for 16 h. Afterward, excess fluorescent dye was removed by dialysis (molecular weight cutoff: 2000 Da) against deionized water for 24 h. The purified Cy5.5-PEG-PLA copolymers were freeze-dried and stored at 4 °C in the dark. Cy 5.5-PEG-PLA copolymers were further assembled with the graft copolymer P(HPMA-Lac-*co*-His)-*g*-PLA and the hydrophobic moiety Dox following the abovementioned procedures, forming Cy-5.5-labeled Dox-loaded polymeric mixed micelles.

The fluorescences of the small molecule free Dox and Cy-5.5-labeled Dox-loaded polymeric mixed micelles (Dox-mixed micelles) (1 μM) were adjusted based on the Dox fluorescence and separately treated with the mouse Lewis lung carcinoma LL/2 cells, which were seeded on coverslips for 24 h at 37 °C with a 5% CO_2_ supply. After 3 and 24 h incubation, the Dox or Cy-5.5-labeled Dox-mixed micelles were removed, following the cells were washed with PBS. The cells were further incubated with the fluorescent dye LysoSensor™ Blue DND-167 for 30 min at 37 °C under 5% CO_2_ conditions. Afterward, excess fluorescent dye was removed and the cells were washed thrice with PBS. For cell fixation, the cells were immersed in 1 mL of 4% paraformaldehyde solution for 10 min. After cell fixation, the cells were washed thrice with PBS and mounted with glycerol on a slide. The mounted samples were observed with the CLSM system at the excitation wavelengths of 373, 488, and 675 nm; and LP filters of 425, 590, and 695 nm were applied to independently measure the fluorescence of the LysoSensor Blue DND-167, Dox, and Cy 5.5.

### 2.9. Biodistributions and Tumor Accumulation

Mouse Lewis lung carcinoma LL/2 cells (1 × 10^7^ cells/mL) were subcutaneously (s.c.) transplanted into the abdomens of 4-week old female BALB/c mice. Two weeks later, when the tumors were approximately 5 mm in diameter, the Cy-5.5-labeled Dox-mixed micelles (5 mg Dox/kg) were administered through the tail vein into the tumor-bearing mice. The mice were anesthetized, and their optical images were acquired using an IVIS imaging system (IVIS 100, Caliper Life Science, PerkinElmer, Waltham. MA, USA) via the Cy 5.5 filter channel at 1, 3, 6, 24, 48, and 72 h post-injection. Additionally, at 6 and 72 h post-administration, the tumor-bearing mice were euthanized and their organs as well as the tumor tissues were collected to evaluate the biodistribution of the Cy-5.5-labeled Dox- mixed micelles.

Dox and Cy-5.5-labeled Dox-mixed micelles (5 mg/kg) were i.v. administered into LL/2 cell-inoculated nude mice, whose tumors were approximately 10 mm in diameter. At 72 h post-injection, the tumor-inoculated mice were euthanized and the tumors, livers, kidney, lung, spleen, and heart were excised. These tumor and organs excised from the mice were further embedded in OCT medium (MICROM) and frozen with dry ice. The excised tumors and organs were prepared into frozen sections (4 μm thick) using a microtome-cryostat (CM3050 S, Leica). Afterward, the frozen tissue sections attached on the slides were treated with 1 mL of methanol at −20 °C for 30 min and washed thrice with PBS. The tissue sections were stained with fluorescent dye, DAPI, and mounted with a cover slip. The tissue sections were scanned using the CLSM system. Excitation wavelengths of 390, 488, and 675 nm; and LP filters of 460, 590, and 695 nm were independently used to detect the fluorescence of DAPI, Dox, and Cy 5.5.

### 2.10. In Vivo Antitumor Activity and Toxicity Assessment

The xenografted tumor-bearing mouse model was established as previously described [22,24]. In brief, 1 × 10^6^ LL/2 cells (0.1 mL) were subcutaneously inoculated into the abdomens of female BALB/c mice. The tumor sizes were measured using a Vernier caliper and the tumor volume (V) was calculated following the formulation: V = (ab^2^)/2, where a and b indicate the major and minor axes of the tumor, respectively. When the transplanted tumor volume reached 50 mm^3^ (approximately 7 d after cell inoculation), the animals were randomly divided into three groups with six mice per group. The mice in each group were independently intravenously administered with PBS (control), 5 mg/kg Dox, or Dox-loaded micelles at a dose of 20 mg/kg of Dox at day 0, 3, and 6. Follow-up was performed for 22 days after administration of the treatments. The tumor sizes and the body weight of the mice were carefully recorded to evaluate the antitumor activity and the toxicity.

To identify the cytotoxicity toward cancer cells in vivo, the LL/2-cell-bearing mice were simultaneously injected with PBS (control), 5 mg/kg of Dox, and Dox-mixed micelles. Fourteen days later, the mice were euthanized and their tumors were excised for tumor apoptosis analysis. The excised tumors were embedded into OCT medium and frozen using dry ice. The tumor tissues were prepared into 4 μm thick frozen sections using a microtome-cryostat and attached to the slides. The frozen sections were further fixed in 4% paraformaldehyde for 20 min at 25 °C. Afterward, the slides were washed with PBS and Tween 20 PBS solutions (containing 0.3% Tween 20 in PBS at pH 7.4) thrice for 10 min. The apoptotic cells were further stained following the instructions for the ApopTag^®^ Fluorescein In Situ Apoptosis Detection Kit S7110, and the nuclei of the cells in tissues were labeled with DAPI. The fluorescence of the apoptotic cells and cell nuclei was detected using a CLSM system with excitation wavelengths of 390 and 488 nm and the appropriate emission wavelength.

## 3. Results and Discussion

### 3.1. Polymer Characterizations

In this study, our polymeric mixed micelles were composed of a diblock copolymer mPEG-*b*-PLA and a graft copolymer (P(HPMA-Lac-*co*-His)-*g*-PLA). The block copolymer was acquired via ring-opening polymerization. The mPEG-*b*-PLA copolymer was characterized using ^1^H-NMR, FT-IR, and GPC, as shown in Figure S1 in the Supporting Information. The ^1^H-NMR spectra (Figure S1a) indicated that there were 24 repeating units of PLA in the copolymer. The FT-IR spectra in Figure S1b display the stretching vibration of the ether groups in the PEG segments at 1110 cm^−1^ and the stretching vibration of the ester groups in the PLA segments at 1750 cm^−1^, representing the successful synthesis of mPEG-*b*-PLA. The GPC results in Figure S1c show that the dispersity of the copolymer is 1.1, indicating the homogeneity of the copolymer. On the basis of these results, we determined that a homogenous diblock copolymer mPEG-*b*-PLA was successfully synthesized.

The other graft copolymer was synthesized via two main polymerization steps: (1) HPMA-PLA synthesis and (2) P(HPMA-Lac-*co*-His)-*g*-PLA synthesis, as shown in Scheme S1 in the Supporting Information. Firstly, the HPMA-PLA was prepared from HPMA and d,l-lactide molecules via ring-opening polymerizations as Figure S2a shows. The HPMA-PLA was characterized using ^1^H-NMR, as shown in Figure S2 in the Supporting Information. The ^1^H-NMR spectra are illustrated in S-2, showing that HPMA-PLA_3_ and HPMA-PLA_6_ were obtained. HPMA-PLA_3_ and HPMA-PLA_6_ were further reacted with various ratios of other HPMA-containing moieties, including HPMA-dilactate, HPMA-Boc His [18,26], and HPMA, under free radical polymerization, whereas HPMA-dilactate was in advance synthesis via a ring-opening reaction, as Figure S3a shows. The chemical structure of HPMA-dilactate were identified using ^1^H-NMR as S-3 illustrates and Figure S3b shows. After the free radical polymerization, the hydrogenated reactions were performed to remove the Boc protective groups, which could protect the active amino group from reacting with carboxylic groups during reaction. The resulting graft copolymers were characterized using ^1^H-NMR and FT-IR, as shown in S-4, Figure S4 and Tables S1 and S2 in the Supporting information. The infeed monomer mole ratios and compositions of these graft copolymers were calculated from the ^1^H-NMR spectrum (Figure S4a) and the results are shown in Table S1. As shown in Table S2, the molecular weight (Mn) of the graft copolymers fabricated from HPMA-PLA_3_ and the ratios of the histidine and dilactate (S1–S5) range from 20,000 to 33,000, whereas the Mn of the graft copolymers comprising HPMA-PLA_6_ and various numbers of the histidine and dilactate (L1–L5) range from 30,000 to 42,000. In their FT-IR spectrum, their characteristic peaks are shown in Figure S3b, including the C=O stretching of amide bonds in histidine at 1660 cm^−1^, the C=O stretching of ester bonds in PLA at 1750 cm^−1^, and the O–H stretching of hydroxyl groups in HPMA at 3400 cm^−1^, identifying the components histidine, lactate, and HPMA.

### 3.2. Preparation and Characteristics of Mixed Micelles

**Figure 1:**
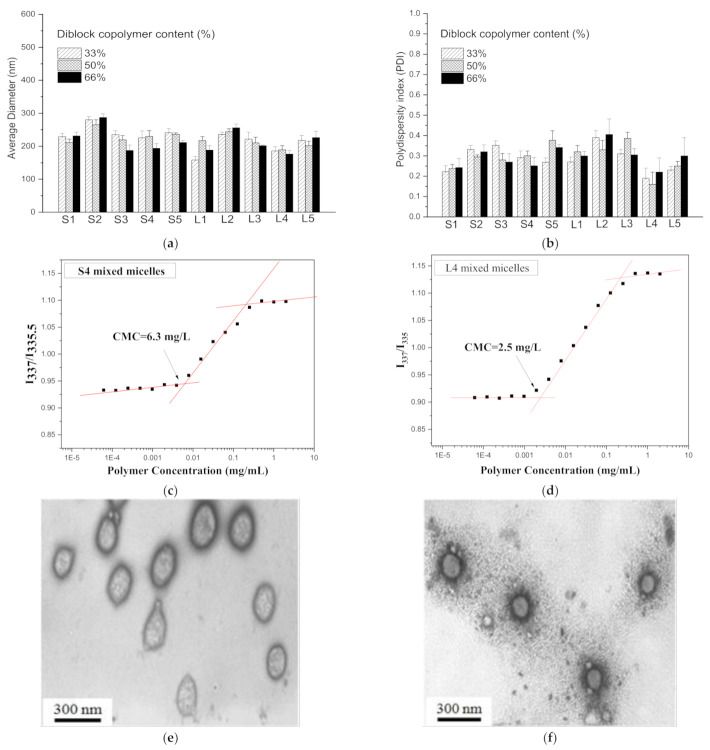
Characterization of the mixed micelles. (**a**) The particle sizes and (**b**) PDI values of the mixed micelles with various weight ratios of the diblock and graft copolymers. The CMC values of (**c**) S4 and (**d**) L4 polymeric mixed micelles, which were determined via a fluorescent pyrene probe. (**e**) S4. and (**f**) L4 polymeric mixed micelles after staining with 2% uranyl acetate were observed using TEM.

The graft copolymer P(HPMA-Lac-*co*-His)-*g*-PLA and the diblock copolymer mPEG-*b*-PLA were assembled into polymeric mixed micelles via dialysis. The ratios of the two polymers significantly affected the physicochemical properties of these polymeric mixed micelles. Therefore, firstly, we assembled the polymeric mixed micelles with various weight ratios of the two polymers and determined their particle sizes and distributions. The graft copolymer comprised various proportions of histidine, dilactate, and PLA, and each of them was independently assembled with mPEG-*b*-PLA in a variety of weight ratios. These mixed micelles were primarily measured with DLS to determine their particle sizes and PDI. As shown in Figure 1a,b, the minimum particle size and the narrow distribution of the mixed micelles is ca. 200 nm. This value was obtained from the mixed micelles composed of the diblock copolymer mPEG-*b*-PLA and graft copolymer S4 or L4 as they were assembled with 66 wt % diblock copolymer and 24 wt % graft copolymer. The CMC of the S4 and L4 mixed micelles was also determined using a hydrophobic fluorescent pyrene probe [30]. The excitation wavelength of the fluorescent pyrene is detected at 335.5 nm in mPEG-b-PLA solutions; as the polymer concentrations in the solution achieve the CMC value, the excitation wavelength shifts to 337.5 nm, as Figure S5 shows. Therefore, the CMC value was determined by the relative excitation intensity at a wavelength of 337.5 nm (I_337.5_) and 335.5 nm (I_335.5_). The ratios of the fluorescent intensity at 337.5 and 335.5 nm (I_337.5_/I_335.5_) for the various S4 and L4 polymer concentrations are presented in Figure 1c,d, and their values are 6.3 and 2.5 mg/L, respectively. The detected CMC values of the diblock and graft copolymers indicated that the two polymers form micelles in aqueous milieus. The micellar morphologies of the S4 and L4 polymeric mixed micelles were observed using TEM after negative staining, shown Figure 1e,f. The PEG hydrophilic layer was observed as stained by a grey color and was spread around the cores. The particle sizes in the TEM images are approximately 200 nm, which is in agreement with the DLS results.

### 3.3. Stability and pH-Responsiveness

**Figure 2:**
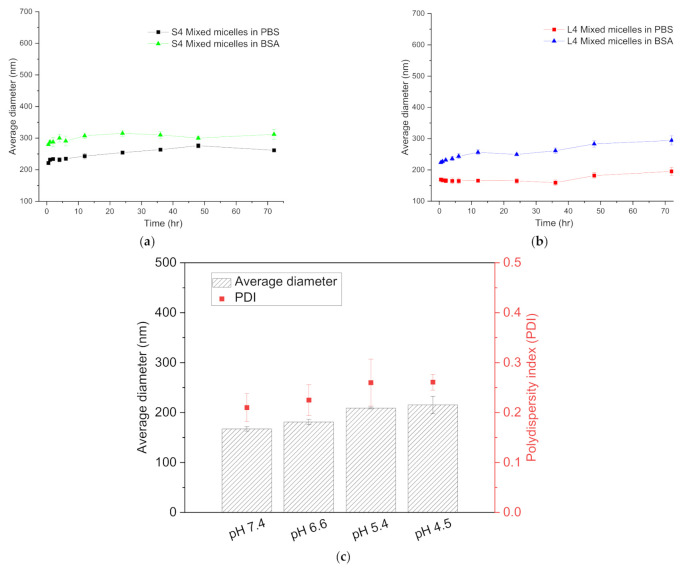
Stability and pH-responsive tests of the mixed micelles. The particle sizes of (**a**) S4 and (**b**) L4 polymeric mixed micelles in pH 7.4 and 4% BSA environments. (**c**) The particle sizes and PDI of the L4 mixed micelles in different pH (pH 7.4, 6.6, 5.4, and 4.5) conditions. The particle sizes and PDI values were determined by dynamic light scattering (DLS). All results are presented as mean ± SD (*n* = 3).

The S4 and L4 mixed micelles were identified as having the smallest particle sizes and micellar structures; the CMC value of S4 mixed micelles was found to be 2.5 times higher than that of L4 mixed micelles. Therefore, the stability of the two mixed micelles was investigated. The S4 and L4 mixed micelles were incubated at 37 °C in PBS to mimic physiological conditions. The particle sizes and distributions of the mixed micelles were monitored for 72 h using DLS. As shown in Figure 2a, when the S4 and L4 polymeric mixed micelles were incubated at 37 °C in pH 7.4 PBS for 72 h, the particle size of both subtly increased. However, Figure 2b indicates that the PDI of the S4 mixed micelles increased 1.3-fold, whereas that of the L4 mixed micelles did not increase even after 72 h incubation. The results showed that, after 72 h of incubation in mimicked physiological conditions, the L4 polymeric mixed micelles maintained their homogeneous particles, displaying their higher stability in comparison to the S4 mixed micelles.

The S4 and L4 polymeric mixed micelles were also incubated at 37 °C in the presence of 4% BSA to imitate the blood environment [31]. The particle sizes and PDI were also monitored for 72 h, as shown in Figure 2a,b. As shown in Figure 2a, the particle sizes of both S4 and L4 polymeric mixed micelles mildly increased in the presence of BSA. However, per Figure 2b, the PDI value of S4 polymeric mixed micelles significantly increased after 72 h incubation, but that of L4 polymeric mixed micelles did not change. The results clearly demonstrated that L4 polymeric mixed micelles exhibit excellent stability in a mimetic blood environment. The high stability of the L4 polymeric mixed micelles is explained by their high content of PLA segments in the graft copolymer P(HPMA-Lac-*co*-His)-*g*-PLA. Hydrophobic PLA segments can be introduced into the interior core of the polymeric mixed micelles, enhancing the hydrophobic interactions of the micellar core. Therefore, the L4 polymeric mixed micelles displayed a lower CMC value and better stability in comparison to the S4 polymeric mixed micelles, which had shorter hydrophobic PLA segments. Notably, even though the L4 mixed micelles demonstrated high stability in mimetic physiological milieus, they still displayed a modest particle size increase after 72 h incubation, due to the degradation of the copolymers. The degradation of the mPEG-*b*-PLA diblock copolymer was previously reported [32]; the degradation of the L4 graft copolymer P(HPMA-Lac-*co*-His)-*g*-PLA was further assessed using GPC in our study. As shown in Figure S4 in the Supporting Information, the molecular weight of the L4 graft copolymer P(HPMA-Lac-*co*-His)-*g*-PLA decreased and its PDI increased during the incubation periods, indicating the degradability of the graft copolymer. However, the degradation was slow; hence, the mixed micelles display enough stability to maintain their structures for at least 72 h.

As mixed micelles are internalized into cells, the pH drops significantly from the physiological value (7.4–7.2) to pH 6.5–5.0 in the endosomes and to roughly pH 5.0 in primary and secondary lysosomes during endocytosis. Additionally, the extracellular pH of tumors (ranging from 6.8 to 6.5) is also slightly more acidic than that of blood and normal tissues [33,34]. Therefore, the pH-responsive behavior of the L4 polymeric mixed micelles was evaluated. First, the L4 polymeric mixed micelles were independently incubated at different pH conditions (pH 4.5–7.4), which imitated the tumor tissue environment (pH 6.6) and the decreasing pH values in endo-lysosomes during internalization (pH 5.5 and 4.5). The particle sizes and PDI values were measured using DLS, as presented in Figure 2c. As shown in Figure 2c, the L4 polymeric mixed micelles displayed increases in particle size and PDI value with decreasing pH values, demonstrating their response to environmental pH. Furthermore, the anticancer drug loading behaviors of the highly stable and pH-responsive L4 polymeric mixed micelles were investigated.

### 3.4. Cytotoxic Assessment

In our study, the potent anticancer drug, Dox, was loaded into the L4 polymeric mixed micelles. In advance, various concentrations of Dox hydrochloride were independently neutralized using a 1.2 molar excess of triethylamine in DMSO to remove the salt form [22,23,24]. To prepare the mixed micelle, a total of 6 mg of the polymers, comprising 33 wt % of graft copolymer L4 and 66 wt % of mPEG-*b*-PLA, was dissolved in the solutions with different drug concentrations, followed by dialysis against Milli-Q water. Afterward, the particle size and PDI) of the mixed micelles were measured using DLS. The Dox-loaded polymeric mixed micelles were freeze-dried and re-dissolved in DMSO to determine the drug content and EE. As illustrated in Table S3 in the Supporting information, when 3 mg/mL of Dox was fed in, the smallest particle size and PDI were detected, which were 165.3 ± 6.3 nm and 0.21 ± 0.06, respectively, whereas the drug contents and loading efficiency were approximately 13% and 40%, respectively. The featured core-shell structures are displayed in Figure S7a in the Supporting Information, demonstrating the formation of the Dox-mixed micelles. Herein, the mixed micelles, loading with 3 mg/mL of Dox were investigated their drug releasing profiles. These Dox-mixed micelles also exhibited pH-dependent drug-releasing profiles, as shown in Figure S7b and completely illustrated in S-7 of the Supporting Information, indicating their intracellular drug-releasing ability [35]. Moreover, the drug-releasing profiles also demonstrated that the mixed micelles can preserve most of their payloads in mimetic physiologic conditions (pH 7.4) for 72 h, demonstrating the high stability of the Dox-mixed micelles and their long circulating potential in the human body.

**Figure 3:**
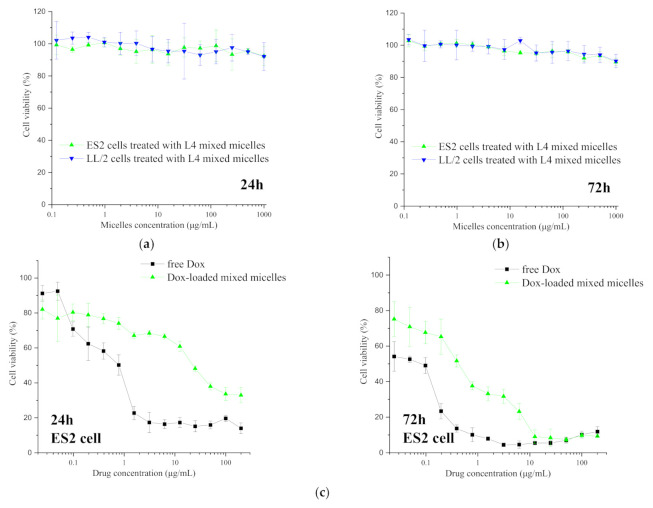

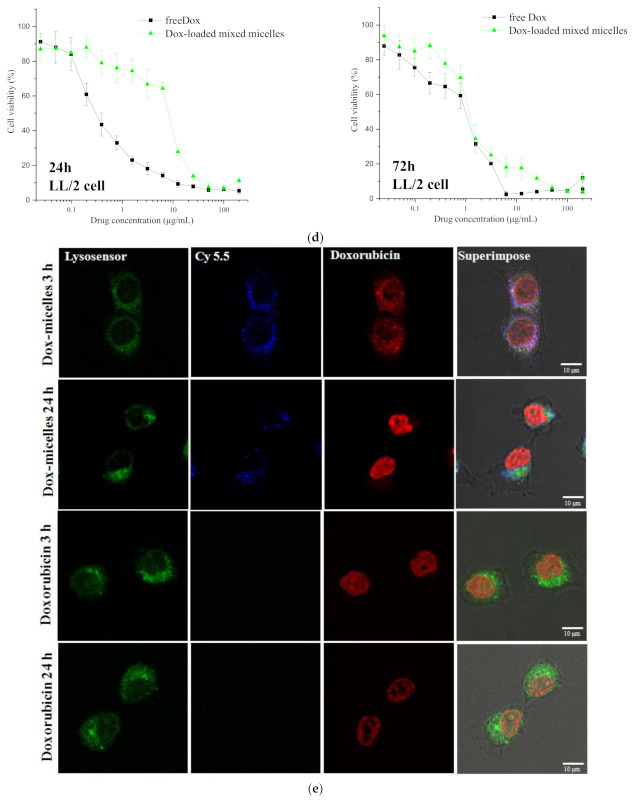
In vitro cytotoxic assessment and internalization observation. The cytotoxicity of the blank L4 polymeric mixed micelles was assessed with human ovary cancer cell line ES2 and mouse Lewis lung cancer cell line LL/2 after (**a**) 24 h and (**b**) 72 h co-incubation. The in vitro toxicity of the free Dox and Dox-mixed micelles toward the (**c**) human ovary cancer cell line ES2 and (**d**) mouse Lewis lung cancer cell line LL/2 were also estimated for 24 and 72 h. The cytotoxicity was determined using an MTT assay. The cell viability is presented as mean ± SD (*n* = 3). (**e**) The free Dox and Dox-mixed micelles, which were labeled in advance with the fluorescent Cy 5.5, were observed using a CLSM system. The LL/2 cells were incubated with Cy-5.5-labeled Dox-loaded micelles and doxorubicin at 37 °C for 3 and 24 h; afterward, their endo-lysosomes were also stained with LysoSensor for observation. The fluorescence of the Cy 5.5, Dox, and LysoSensor were independently detected with the appropriate excitation and emission wavelengths and are presented in blue, red, and green, respectively.

Long-circulating nanocarriers were reported to sensitize the cancer cells toward their payloads [36]. Herein, the highly stable and pH-responsive Dox-mixed micelles were applied to treat cancer cells that display relatively low therapeutic sensitivity toward Dox. The low sensitivity of human ovary cancer cell line ES2 and murine mouse Lewis lung cancer cell line LL/2 toward the antineoplastic reagent Dox was reported [37,38,39,40], so both of them were separately incubated for the small molecular Dox (free Dox) for 24 and 72 h to track their cell inhibitory effects. The cell inhibitory effects were determined using an MTT assay, and the results are shown in Figure 3 and Table S4 in the Supporting Information. As depicted in Figure 3a,b, the blank mixed micelles displayed high cell viability toward the two cell lines after 24 and 72 h co-incubation, indicating their high biosafety. However, when the cancer cells were treated with sequential concentrations of the Dox or Dox-mixed micelles, whose concentrations were adjusted based on the levels of Dox, a time- and dose-dependent effect was observed. As Figure 3c shows, the cell viability of the Dox and Dox-mixed micelle toward ES2 cells decreased over the incubation periods. However, per Table S4, the IC_50_ values of the ES2 cells treated with free Dox were still lower than those of the cells treated with Dox-mixed micelles due to the distinct mechanisms of free Dox and Dox-mixed micelle internalization. Small molecules can freely diffuse and penetrate into cancer cells, whereas Dox-mixed micelles display a slow and complex endocytic process [41,42]. Higher IC_50_ values were found in the LL/2 cells treated with Dox-mixed micelles for 24 h (Figure 3d and Table S4). However, the IC_50_ values and cell inhibitory trends of the LL/2 cells treated with free Dox approximated those of the cells treated with Dox-mixed micelles, as shown in Figure 3d and Table S4. These findings are due to the sensitization of the Dox-mixed micelles toward LL/2 cells; even the Dox-mixed micelles were slowly internalized. Therefore, we focused on the therapeutic efficiency of Dox-mixed micelles toward the LL/2 murine lung cancer cell line.

### 3.5. Observation of the Intracellular Drug-Releasing Behaviors in Cancer Cells and Internalization

The internalization of the Dox-mixed micelles into the murine Lewis lung cancer cell line LL/2 was observed. As we previously mentioned, the Dox-mixed micelles could release their embedded Dox in mimetic endo-lysosomes during endocytosis (pH 5.4 and 4.5); their intracellular Dox-releasing behaviors as well as the internalization process were further observed using CLSM. Firstly, the Dox-mixed micelles were tagged with a Cy 5.5. fluorescent dye. In advance, the amine-capped PEG-PLA was fabricated from Boc-NH-PEG (Mw 4750) and d,l-lactide by ring-opening polymerization [22,24]. The Boc protective groups in the former enable to prevent the by-product made from the amine groups and the carboxylic groups of the lactate during ring opening polymerization. Therefore, after hydrogenation to remove the Boc-protective groups, amine-capped PEG-PLA was obtained. NH_2_-PEG-PLA was further reacted with the fluorescent dye Cy 5.5 for 24 h. After removing the excess fluorescent dye, the acquired Cy 5.5-PEG-PLA, whose weight-average molecular weight (Mw) and PDI values are 5930 and 1.13, respectively, was assembled with L4 graft copolymer and Dox, following the above-mentioned methods for mixed micelles preparation. The prepared Cy 5.5-labeled Dox-mixed micelles and the free Dox were co-cultured with LL/2 murine lung cancer cells for 3 and 24 h, respectively, and the endo-lysosomes of the cells were stained with LysoSensor.

The CLSM images are shown in Figure 3e and the visualized fluorescence of the endo-lysosome, Cy 5.5, and Dox, are presented in green, blue, and red, respectively. Figure 3e indicates that the Dox (indicated in red) penetrated into the cell nuclei in 3 h, whereas the blue florescence of the Cy-5.5-labeled Dox-mixed micelles was observed overlapping the green fluorescence, demonstrating that the Dox-mixed micelles were located into the endo-lysosomes. At 24 h post-incubation, the Dox fluorescence (presented in red) was observed permeating into the cell nuclei of the cells treated either with the free Dox or the Dox-mixed micelles. The CLSM images verified the cytotoxic tests of the Dox and Dox-mixed micelles with LL/2 cells, providing strong evidence that LL/2 cells took up the micelles and released them within the cells. Free Dox can enter cells and penetrate cell nuclei by diffusion, therefore causing abrupt cytotoxicity; Dox-mixed micelles were taken up by the LL/2 cells, releasing their loaded Dox in endo-lysosomes; thereby, the released Dox permeated into the cell nuclei to toxify the cells.

### 3.6. In Vivo Bio-Distribution and Tumor Accumulation

**Figure 4:**
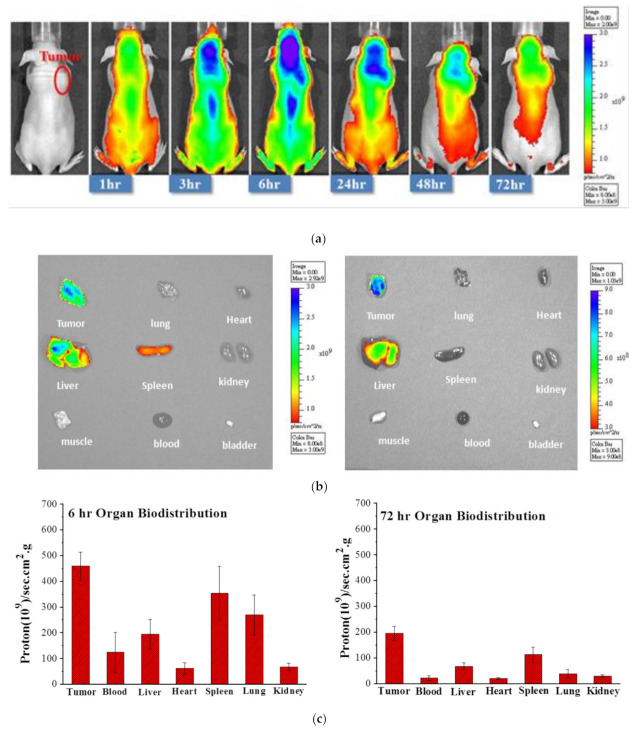
Tumor accumulation and biodistribution of the Dox-loaded mixed micelles. The LL/2 mouse Lewis lung cancer cells were subcutaneously inoculated into nude mice. As the tumor size grew to 5 mm in diameter, the mice were intravenously injected with Cy-5.5-labeled Dox-loaded mixed micelles. The fluorescence of the Cy-5.5-labeled Dox-mixed micelles was detected using IVIS. (**a**) In vivo optical fluorescence imaging of LL/2 tumor xenografted nude mice, which were treated with Cy-5.5-labeled Dox-loaded mixed micelles. (**b**) At 6 and 72 h post-injection, the tumor-bearing mice were euthanized, and the tumor tissues and organs were harvested. The photoimaging was performed and the fluorescence of the tumors and organs was detected to assess the biodistribution of the mixed micelles. (**c**) The proton intensities of the tumors and organs were also analyzed.

Dox is an effective antineoplastic drug, but its lack of tumor selectivity causes severe side effects in the clinic, such as cardiotoxicity [43]. The administration of Dox in nanoparticle-associated form has been advocated as a method to reduce the acute side effects of the drug [22,44] by precise tumor accumulation. To determine the tumor-accumulating ability of our Dox-loaded micelles in vivo, BALB/c nude mice bearing mouse Lewis lung carcinoma LL/2 cells were treated with one injection of Cy-5.5-labeled Dox-mixed micelles, and the optical fluorescence was tracked over time using IVIS. The live animal imaging in Figure 4a shows that the relative fluorescence intensity in tumors increased gradually at 1, 3, 6, and 24 h after intravenous injection, indicating that our Cy-5.5-labeled Dox-mixed micelle preferentially accumulates into tumor tissues. The relative fluorescence intensity of the Cy-5.5-labeled Dox-mixed micelle remained in the mouse body until 72 h after intravenous injection, demonstrating the long-circulating property of our mixed micelles. At 6 and 72 h post-administration, the LL/2-inoculated mice were euthanized; the tumor and organs, including the liver, lung, heart, spleen, kidney, muscle, blood, and bladder, were dissected; and their fluorescence was detected. The ex vivo fluorescence and proton intensity of the tumor and each organ are presented in Figure 4b,c, respectively. The ex vivo images in Figure 4b show that 6 h after i.v. administration, strong fluorescence was observed mainly in the tumor, liver, and spleen. Figure 4c also indicates that the proton intensity in the tumor was approximately 1.3-fold higher than in the spleen and 1.5-fold higher than in the liver. At 72 h post-injection, the fluorescence was only detected in the tumor and liver, as shown in Figure 4b. Notably, the fluorescence in the tumor was stronger than in the liver. The proton intensity in Figure 4c decreased in the tumor and all the organs 72 h after administration due to the degradation and metabolism of the Dox-mixed micelles. However, Figure 4c demonstrates that the protons were mainly detected in the tumor and their intensity was at least 1.5- and 2-fold higher than in the spleen and liver, respectively.

**Figure 5:**
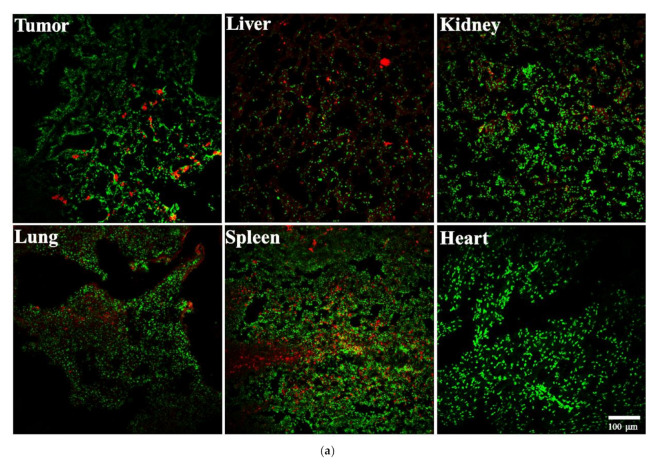

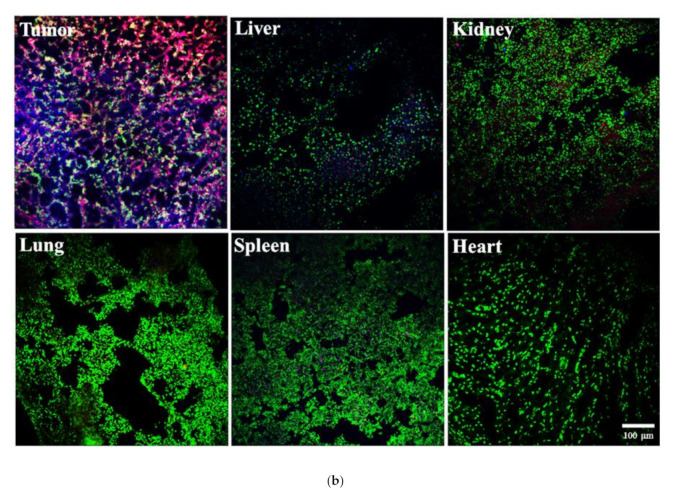
Confocal images of tumors and organs harvested from the mice treated with (**a**) doxorubicin and (**b**) Cy-5.5-labeled Dox-mixed micelles after 72 h i.v. administration. The fluorescence of the doxorubicin and Cy 5.5 is denoted using red and blue, respectively. The cell nuclei were also stained with DAPI and are presented in green.

To further investigate the drug release, the middle parts of the tumor tissues and the organs collected from the mice treated with Cy-5.5-labeled Dox-mixed micelles at 72 h post-injection were prepared into frozen sections and their fluorescence, including Cy 5.5, Dox, and the DAPI-stained cell nuclei, were independently detected using CLSM, presented in blue, red, and green in Figure 5, respectively. Simultaneously, 5 mg/mL of free Dox was also i.v. administered into LL/2-inoculated nude mice. At 72 h post-injection, the mice were euthanized, and the tumors and organs were collected and prepared into frozen sections. The Dox fluorescence was also detected using CLSM to study the tumor accumulation and drug-releasing behaviors of the small molecule, Dox. As shown in Figure 5a, 72 h post-treatment with Dox, Dox fluorescence could be detected in most organs (presented in red) and mild Dox fluorescent intensity in tumors. However, Figure 5b shows that 72 h after i.v. administration, an intense Cy 5.5 fluorescence (indicated in blue) was observed deposited in the tumor lesion, indicating the longevity and the significant accumulation of the Cy-5.5-labeled Dox-mixed micelles. Furthermore, the red fluorescence of Dox in tumor lesions overlapped with the cellular green fluorescence, indicating that the Dox was released within the cells and even penetrated the cell nuclei. Dox fluorescence (denoted by red) and Cy 5.5 fluorescence (denoted by blue), however, was not displayed by the liver, kidney, lung, heart, or other tissues at 72 h post-injection, clearly demonstrating that Dox-mixed micelles preferentially and mainly accumulate in tumors when circulating, whereas they did not accumulate or release their payload in other organs. These ex vivo images also clearly illustrate the tumor selectivity of the Dox-mixed micelles.

### 3.7. In Vivo Antitumor Activity

To evaluate the antitumor efficacy of the Dox-mixed micelles, the mouse Lewis lung cancer LL/2 cells borne by nude BALB/c mice were separated into three groups and the mice in each group were independently treated with saline solution (control group) or 5 mg/mL of Dox and Dox-mixed micelles, whose dosage was previously adjusted based on the Dox concentration to 20 mg/mL. We used a lower dosage of the free Dox because the high dosage of the Dox resulted in low survival rates (data not shown). All of the treatment modalities were administered by intravenous injection at a frequency of thrice with a 3 day interval when the tumors reached a volume of 0.5 cm^3^. The tumor volume and the mice weights were monitored to evaluate the tumor inhibition and the in vivo toxicity, respectively; the survival rate per group was recorded.

**Figure 6:**
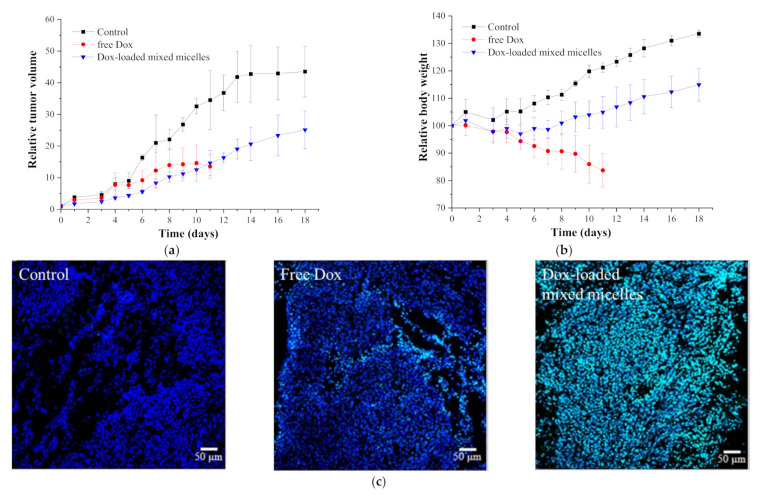
In vivo antitumor efficiency and toxicity evaluation. (**a**) In vivo anticancer efficacy and (**b**) body weight changes in LL/2 mouse Lewis lung cancer cells borne by BALB/c nude mice detected after intravenous administration of saline (control), 5 mg/kg doxorubicin (free Dox), and 20 mg/kg Dox-loaded mixed micelles at day 0, 3, and 6. (**c**) TUNEL apoptotic cell detection of tumor cells in tumor-bearing mice 14 days after intravenous administration of saline, 10 mg/kg of free doxorubicin, and Dox-loaded mixed micelles. Green, apoptotic cells; blue, DAPI.

The tumorigenicity of the Lewis lung carcinoma cell line LL/2 was previously reported. Bertram et al. indicated that a 0.1 g increase in the subcutaneous tumor mass needs less than 23 h and that subcutaneous LL/2 cells require only 40 h to gain 1 g in weight [45]. In our study, the tumor size of the mice treated with saline (control) increased rapidly and the relative tumor volume displayed a ca. 41.8-fold increase after 13 days, as shown in Figure 6a. Tumor volumes with treatment with Dox-mixed micelles showed a slow increase: 25.1-fold after 18 days. This clearly shows that Dox-mixed micelles exhibited considerably higher antitumor activity compared to the control. Mice that were treated with Dox also exhibited a slow increase (13.5-fold) after 11 days. However, as shown in Figure 6b, the mice’s weight significantly decreased after Dox injection. This result indicates the severe toxicity due to Dox injection. Conversely, as shown in Figure 6b, the mice’s weight increased after treatment with saline solution and Dox-mixed micelles. The mice in control group gained weight (33.5%) after the 22 day follow-up mainly because of the rapidly increasing tumor volumes, whereas the mice with Dox-mixed micelles displayed subtle weight gain because the Dox-mixed micelles efficiently inhibited tumor growth while significantly reducing doxorubicin’s toxicity. This increased in vivo antitumor activity of Dox-mixed micelles is attributed to longevity, tumor-specific accumulation, and effective intracellular release in cancer cells.

### 3.8. Tumor Apoptosis

To further assess the cytotoxicity of the Dox-mixed micelles in tumor tissues, the LL2-cell-inoculated BALB/c nude mice were divided into three groups and saline (control), 10 mg/mL of Dox, or Dox-mixed micelles, the dosage of which was adjusted based on the Dox concentration were injected via the tail veins into the mice in each group. After an 18 day follow-up, the mice were euthanized and the tumor tissues of the mice in each group were collected. Afterward, the tumor tissues were prepared into biopsy specimens with TUNEL staining to assess the apoptosis of cancer cells; the nuclei of tumor cells were stained by DAPI. The biopsy specimens with staining were observed using CLSM and are shown in Figure 6c. In Figure 6c, the apoptotic cells are exhibited in green and the cell nuclei in blue. The CLSM images of the biopsy specimens from the control groups did not display any green, indicating that tumor cells from the mice treated with saline did not undergo significant apoptosis. In the CLSM images of the biopsy specimens from the mice treated with free Dox, we observed only a little mild green color in tumor tissues, revealing that the free Dox was able to induce apoptosis, while its rapid clearance resulted in a limited effect. However, the CLSM images of the tumor tissues from the mice treated with Dox-mixed micelles displayed strong green fluorescence. These TUNEL results clearly demonstrate the superior antitumor efficiency of the Dox-mixed micelles in vivo (Figure 6a). The highly stable and pH-responsive Dox-mixed micelles are able to be transported into tumor tissue and release Dox in the cells, resulting in an enhancement in apoptosis toward cells in tumor tissues.

## 4. Discussion

Dox is one of the most effective anthraquinone drugs, commonly used against a wide range of cancers. Despite doxorubicin’s potency, its clinical use causes severe side effects, such as cardiotoxicity [43]. Additionally, Dox sensitivity varies between the different cell types and it can be downregulated with sustained treatment [46,47,48]. The administration of Dox in nanoparticle-associated form has been advocated as a method to reduce the acute side effects of the drug [22,44] and to sensitize Dox in anticancer therapeutics [36,49]. However, any nano-sized drug delivery system, such as polymeric micelles, must specifically accumulate in cancer lesions and rapidly release their payloads [4,50,51]. As suggested by Maeda et al., a long-circulating nanocarrier, which persists in vivo over 24 h has an ability to passively deliver and selectively target designed nanostructures to tumors within the size range of 7–400 nm and accumulate within solid tumors [2,52,53,54]. It can also maintain the blood level of its loaded drug, thereby enhancing the therapeutic effect of the drug because interactions in the targeted organ are prolonged [55,56]. Furthermore, Krishna et al. reported that long-circulating carriers deliver between 3 and 10 times more drug to solid cancerous lesions than the drug in its free state, thereby significantly increasing the sensitivity and the therapeutic efficiency toward anticancer drugs [6]. In our study, we fabricated a highly stable polymeric mixed micellar system, comprising a diblock copolymer mPEG-*b*-PLA and a graft copolymer P(HPMA-Lac-*co*-His)-*g*-PLA, to carry the anticancer drug doxorubicin. As suggested by Kataoka et al., the stability of polymeric micelles can be improved by decreasing their CMC since a low CMC inhibits the dissociation of any copolymer from micelles and hence improves the micellar stability [4,28]. Therefore, polymeric micelles with a lower CMC have the ability to maintain their intact micellar structures and prevent the leakage of drugs after blood dilution in the physiological environment [28]. The CMC value of polymeric micelles can be reduced by increasing the physical interactions of the hydrophobic segment [57,58]. In our study, we elongated the hydrophobic segments of the graft copolymers, thereby reducing the CMC and increasing the stability in mimetic physiological conditions, as shown in Figure 2a,b. In the presence of BSA, since the hydrophilic surface, PEG provides steric stabilization from protein adsorption and the low CMC of the mixed micelles, the mixed micelles remain stable in particle size and distribution. Therefore, in Figure 4, the highly stable Dox-mixed micelles show their selective tumor deposits in LL/2-cell-bearing nude mice. Their longevity allowed their tumor accumulation even 72 h after injection.

To achieve rapid drug release in targeted tissues or cells, a stimuli-responsive nanocarrier was fabricated. A pH-responsive nanocarrier can respond to the mildly acidic tumor tissue environment (pH 6.5–6.8) and acidic endosomes (pH 5–6) or lysosomes (pH 4–5) environments during internalization; therefore, it has been considered a good option to employ in anticancer treatment [23,59]. In comparison to the traditional polymeric micelles comprising only one kind of polymer, the mixed micelles composed of more than two polymers can easily achieve their pH functionality by introducing a pH-responsive polymer [2]. In our study, histidine molecules were appended to the graft copolymer to achieve pH-responsiveness [60]. In Figure 2c,d, the mixed micelles display significant particle size and particular distribution increases in pH 5.4 and 4.5 due to the protonation of the histidine molecules. The pH-responsiveness also facilitates the release of the loaded Dox at pH 5.4 and 4.5, as shown in Figure S7b in the Supporting Information. The drug-releasing behavior also occurs in the interior of cancer cells (Figure 3e).

The highly stable and pH-responsive Dox-mixed micelles can be selectively transported into cancer lesions and release their payloads, mainly in an intracellular manner. These effects clearly reflect their superior antitumor efficiency (Figure 6a,c) and their low toxicity (Figure 6b) in vivo. Most importantly, these polymeric materials, including the mPEG-*b*-PLA and the graft copolymer P(HPMA-Lac-*co*-His)-*g*-PLA, are highly biocompatible and degradable (Figure S6) [32]; hence, the biosafety of the mixed micelles is high (Figure 3a). The mixed micelles, comprising a diblock copolymer mPEG-*b*-PLA and a graft copolymer P(HPMA-Lac-*co*-His)-*g*-PLA, have potential application as a drug delivery system in anticancer treatment.

## 5. Conclusions

In this study, we prepared polymeric micelles with enhanced stability using a simple mix of graft and diblock copolymers. All the polymeric materials and the mixed micelles in this study are relatively safe. The cytotoxicity values and confocal images for mouse Lewis lung carcinoma LL/2 cells showed that the mixed micelles deliver Dox efficiently to the nuclei of cells in vitro. The in vitro results also showed that in comparison to free doxorubicin, our carrier efficiently enhances the intracellular drug release and hence sensitizes cancer cells toward Dox. Our mixed micelle specifically delivers drugs to tumors in vivo, with restrictive particle extravasation to other normal organs. In vivo experiments clearly showed that animals treated with long-circulating polymeric micelles exhibited significantly increased inhibition of the growth of mouse Lewis lung tumors. We observed that long-circulating polymeric micelles have considerable potential for use in cancer therapy to address the issue of drug resistance.

## Data Availability

Data can be requested upon reasonable request.

## Supplementary Materials

The following are available online at https://www.mdpi.com/article/10.3390/cancers13153816/s1, Scheme S1: Chemical synthesis route of P(HPMA-Lac-*co*-His)-g-PLA; Figure S1: Characterization of mPEG-*b*-PLA; Figure S2: Synthesis and characterization of HPMA-PLA; Figure S3: Synthesis and characterization of HPMA-dilactate; Figure S4: Characterization of P(HPMA-Lac-*co*-His)-g-PLA; Figure S5: Fluorescence spectra of 6 × 10^−6^ M pyrene in different mPEG-*b*-PLA copolymer concentrations (mg/L); Figure S6: Degradation of the graft copolymers; Figure S7: The morphology and drug-releasing profile of the Dox-loaded mixed micelles; Table S1: The composition of P(HPMA-Lac-*co*-His)-g-PLA graft copolymer; Table S2. The average molecular weight of P(HPMA-Lac-*co*-His)-g-PLA graft copolymer; Table S3: Particle size, polydispersity index (PDI), encapsulation efficiency (EE), and loading capacity (LC) of doxorubicin-loaded mixed micelles (polymer concentration was 6 mg/mL); Table S4: IC_50_ values for free doxorubicin, G4, and G9 Dox-loaded micelles for cancer cells.
