# Supplementary material for: Doxorubicin-Loaded Mixed Micelles Using Degradable Graft and Diblock Copolymers to Enhance Anticancer Sensitivity
Source: Cancers (Basel). 2021 Jul 29;13(15):3816. doi: 10.3390/cancers13153816 (PMC8345050; doi:10.3390/cancers13153816)
Supplement: Supplementary file 1 [file cancers-13-03816-s001.zip › cancers-1314747-supplementary.pdf]

Supplementary Materials

Doxorubicin-Loaded Mixed Micelle Using Degradable Graft and Diblock Copolymers to Enhance Anticancer Sensitivity

Yi-Chun Chen[a], Chang-Jung Chang[b], Ging-Ho Hsiue[b,c*] and Yi-Ting Chiang[d*]

[a] Department of Forestry, National Chung Hsing University, Taichung City 402, Taiwan (R.O.C.); chenyc@nchu.edu.tw

[b] Department of Chemical Engineering, National Tsing Hua University, Hsinchu, Taiwan, 300 (R.O.C.); ghhsiue@mx.nthu.edu.tw

[c] Department of Chemical Engineering, National Chung Hsing University, Taichung City 402, Taiwan (R.O.C.); kymco741120@hotmail.com

[d] School of Pharmacy, China Medical University, Taichung City 406, Taiwan (R.O.C.); ytchiang@mail.cmu.edu.tw

* Corresponding authors

E-mail: ghhsiue@mx.nthu.edu.tw (G-H Hsiue); ytchiang@mail.cmu.edu.tw (Y-T Chiang)

Supporting Information

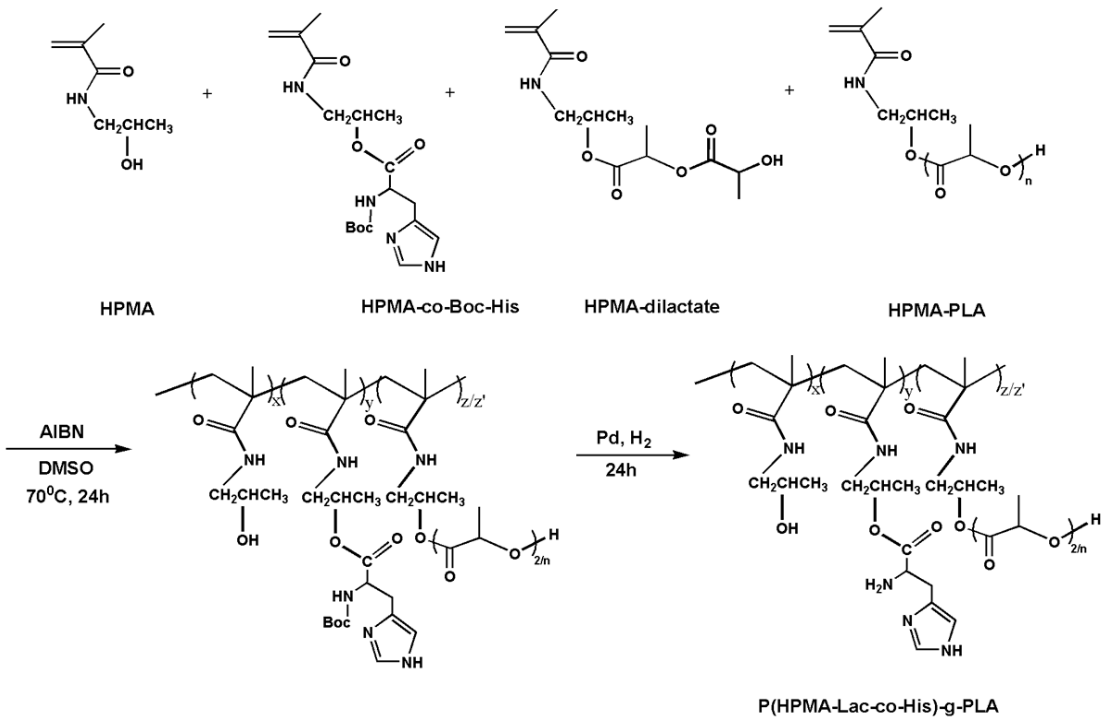

Scheme S1. Chemical synthesis route of P(HPMA-Lac-co-His)-g-PLA.

S-1. Characterization of mPEG-*b*-PLA.

(a)

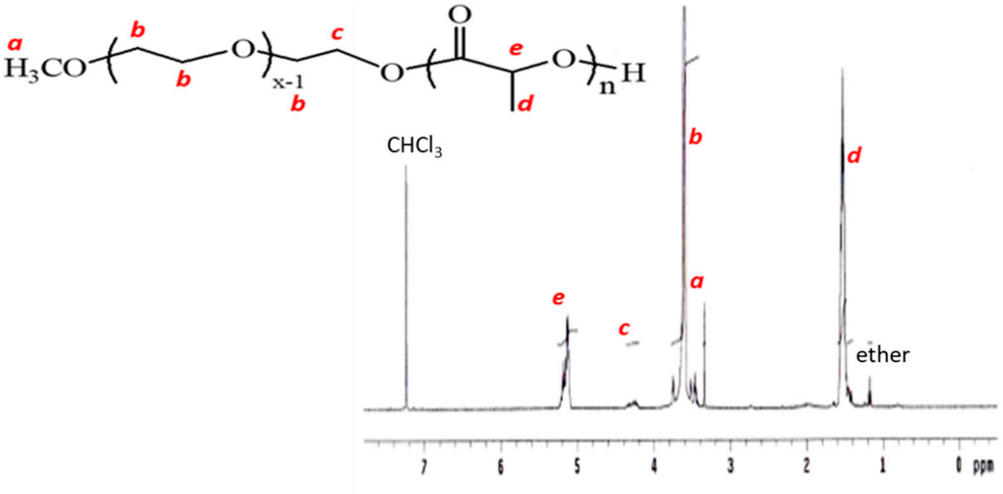

(b)

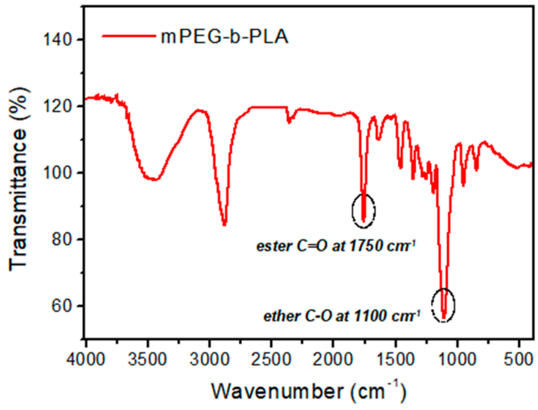

(c)

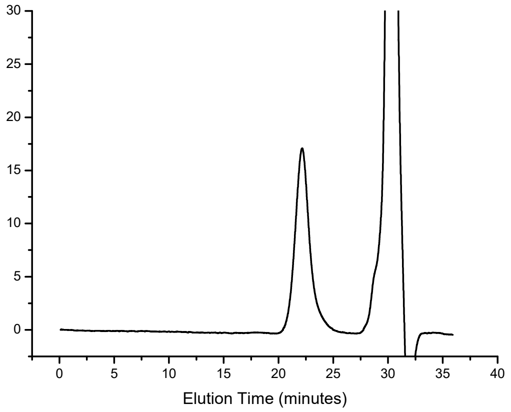

Figure S1. Characterization of mPEG-*b*-PLA. (a) The ^{1}H-NMR spectrum in $CDCl_3$, (b) FT-IR spectrum, and (c) GPC spectrum of mPEG-b-PLA, whose dispersity was determined via the ratios of the weight average molecular weight (Mw) and number average molecular weight (Mn), calculated via the polystyrene standards.

S-2. Synthesis and characterization of HPMA-PLA

The ^{1}H-NMR spectra of HPMA-PLA: δ1.4-1.7 (m, CH_3 from PLA); δ1.92 (s, CH_3 from HPMA); δ 2.2 (s, OH from PLA); δ3.2-3.7 (s, CH_2 from HPMA); δ4.2 (s, CH from HPMA); δ4.8-5.3 (m, CH from PLA); δ5.7, δ6.2 (s, =CH_2 from HPMA).

(a)

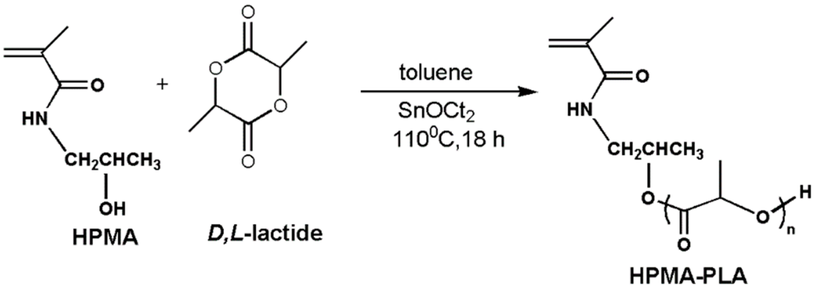

(b)

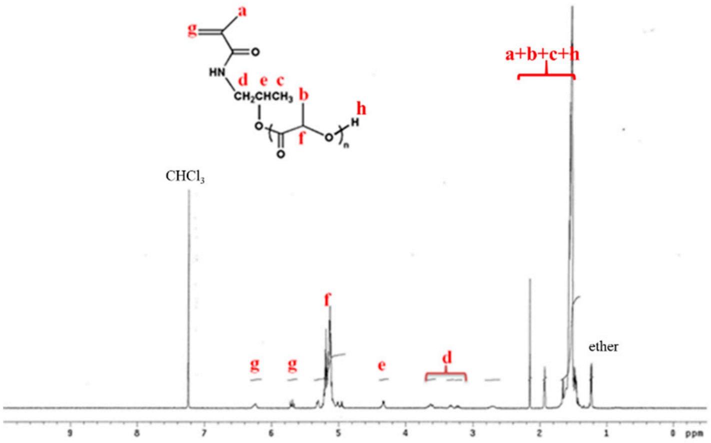

Figure S2. Synthesis and characterization of HPMA-PLA. (a) Chemical synthesis route of HPMA-PLA. (b) The ^{1}H NMR spectrum of HPMA-PLA in $CDCl_3$.

S-3. Synthesis and characterization of HPMA-dilactate

The ^{1}H-NMR spectra of HPMA-dilactate: δ1.2 (s, CO-CH(C*H₃*)-OH from *D,L*-Lactide)；δ1.4-1.6 broad(m, CO-CH(C*H₃*)-O- from *D,L*-Lactide)；δ1.9(s, CH_2=C-C*H₃* from HPMA)；δ2.3(s, CO-CH(CH_3)-O*H* from *D,L*-Lactide)；δ3.2-3.6 broad(m, HN-C*H₂*-CH(CH_3) from HPMA)；δ4.0(s, CH_2-C*H*(CH_3)-OH from HPMA)；δ4.3(s, CH_2-C*H*(CH_3)-O- from HPMA)；δ4.9-5.2 broad(m, CO-C*H*(CH_3)-O- from *D,L*-Lactide)；δ5.3-5.4, δ5.7-5.8(d, C*H₂*-C-CH_3 from HPMA)；δ6.3(s, CO-N*H*-CH_2 from HPMA)。

(a)

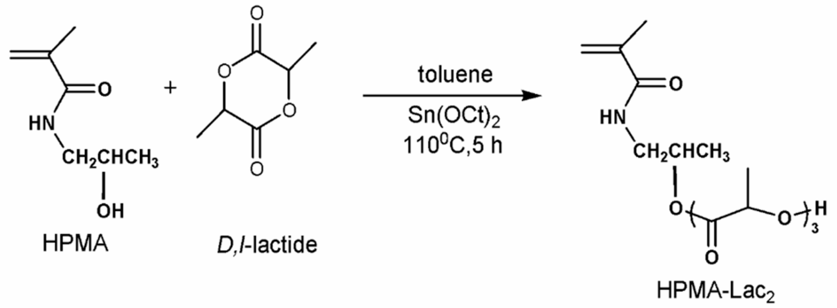

(b)

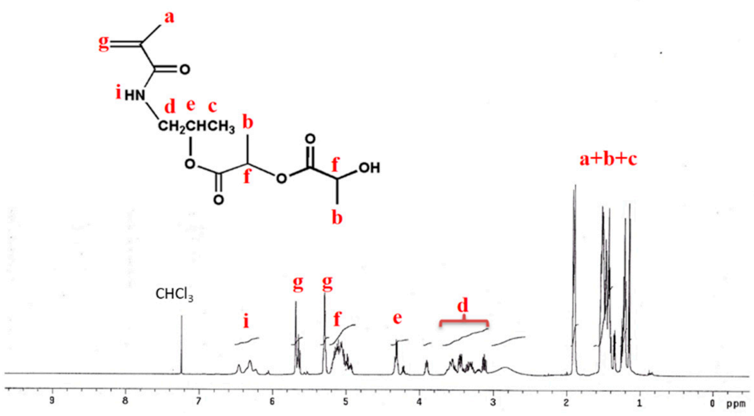

Figure S3. Synthesis and characterization of HPMA-dilactate. (a) Chemical synthesis route of HPMA-PLA. (b) The ^{1}H NMR spectrum of HPMA-PLA in $CDCl_3$.

S-4. Characterization of P(HPMA-Lac-*co*-His)-*g*-PLA

The P(HPMA-Lac-*co*-His)-*g*-PLA product was characterized using ^{1}H-NMR. ^{1}H-NMR (DMSO-d_6, ppm): δ6.9-7.4 board (CO-N*H*-CH_2 from HPMA; Boc-HN-CH(COO) from histidine; CH2-N*H*-CH_2 from histidine); δ7.5 and δ6.8 (C*H* from histidine); δ4.6-5.7 board (CO-C*H*(CH_3)-O- from *D,L*-lactide); δ4.1-4.2 (-CH_2-C*H*(CH_3)- OH) from *D,L*-lactide from PLA and dilatate); δ3.7 (*H*N-CH_2-CH(CH_3) from HPMA; *H*N-CH_2-CH(CH_3) from histidine; δ2.9-3.2 board (CH_2-CH_2 from HPMA; CO-CH_2-CH_2-CH_2-CH_2-C(CN)(CH_3) from ABCPA); δ1.8 (CO-CH(CH_3)-O*H* from *D,L*-lactide); δ1.5, δ1.4, and δ1.3 (C-CH_2-CH_2 from HPMA; CO-CH_2-CH_2-CH_2-CH_2-C(CN) (CH_3 from ABCPA); δ1.1-0.8 board (CH_2-C(CN)(CH_3)-CH_2 from ABCPA; CO-CH(CH_3)-O- from *D,L*-lactide; CO-CH(CH_3)-OH from *D,L*-lactide).

(a)

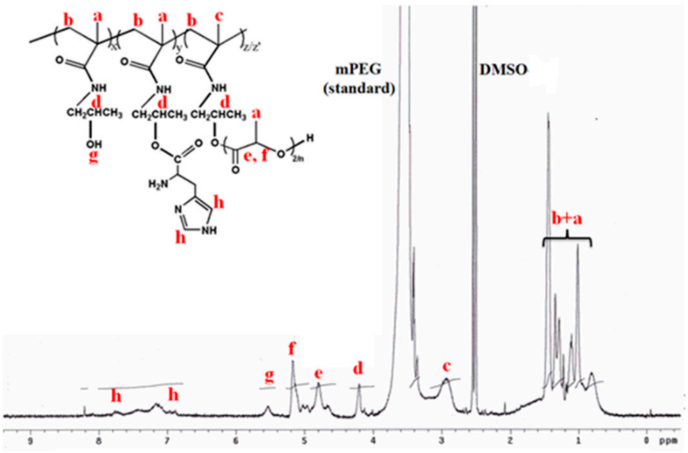

(b)

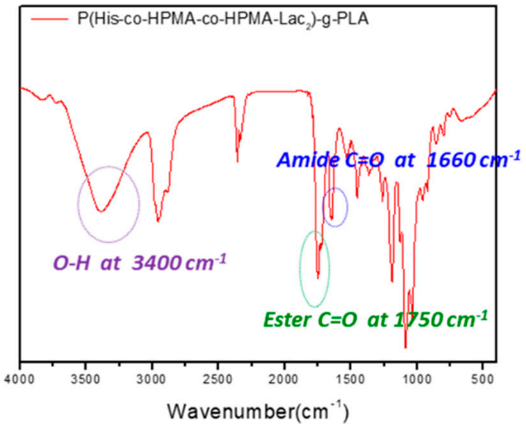

Figure S4. Characterization of P(HPMA-Lac-*co*-His)-*g*-PLA. (a) The ^{1}H NMR spectrum of P(HPMA-Lac-*co*-His)-*g*-PLA in DMSO-d_6. (b) The FT-IR spectrum and (c) the GPC spectrum of P(HPMA-Lac-*co*-His)-*g*-PLA.

Table S1. The composition of P(HPMA-Lac-*co*-His)-*g*-PLA graft copolymer.

Code	Infeed (mole %)				In copolymer (mole %)			
	PLA	Histidine	Dilactate	HPMA	PLA	Histidine	Dilactate	HPMA
S1		0	24.1	72.3	3.4	0	23.2	73.4
S2		9.6	0	86.8	3.1	9.1	0	87.8
S3	3.6	9.6	8.7	76.3	2.8	8.7	9	79.4
S4		9.6	21.7	65.1	2.6	11.7	21.2	64.6
S5		9.6	43.4	43.4	3.6	8.3	43.7	41.1
L1		0	23.2	69.7	6.3	0	24.3	68.8
L2		9.3	0	83.7	6.3	9.1	0	84.6
L3	7	9.3	8.4	75.3	6.8	10	10.2	72.9
L4		9.3	20.9	62.8	6.8	9.5	20.1	63.6
L5		9.3	41.9	41.9	5.9	8.2	40.9	45

* The compositions were characterized based on the ^{1}H-NMR results.

Table S2. The average molecular weight of P(HPMA-Lac-*co*-His)-*g*-PLA graft copolymer.

Code	Mn of backbone	Mn of side chain	Total Mn
S1	22181	6834	29015
S2	14056	6131	20187
S3	15261	6078	21339
S4	20199	6585	26784
S5	26708	6998	33706
L1	23043	13473	36516
L2	16038	12659	28697
L3	19693	11931	31624
L4	20756	12960	33716
L5	28351	13656	42007

* The compositions were characterized based on the ^{1}H-NMR results.

S-5. Fluorescence spectra of pyrene in mPEG-b-PLA copolymer solution

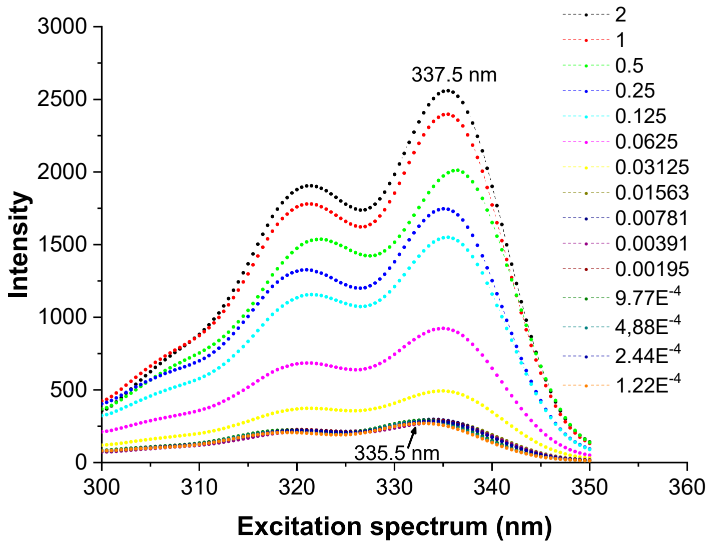

Figure S5. Fluorescence spectra of 6×10^{-6}M pyrene in different mPEG-b-PLA copolymer concentrations (mg/L).

S-6. Degradation of the graft copolymers.

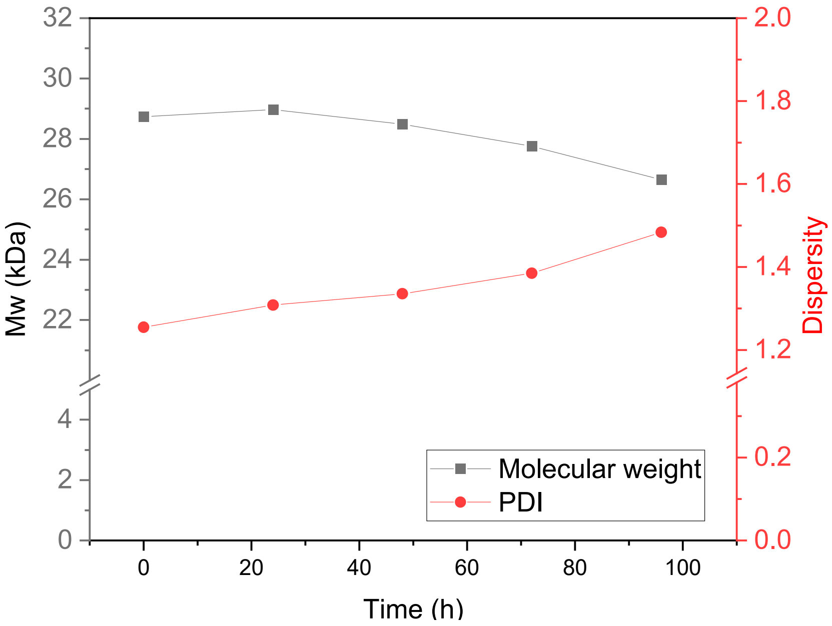

Figure S6. Degradation of the graft copolymers. The L4 graft copolymer P(HPMA-Lac-*co*-His)-*g*-PLA was incubated at pH 7.4 for 96 h. The molecular weight and the dispersity were detected using GPC.

Table S3. Particle size, polydispersity index (PDI), encapsulation efficiency (EE), and loading capacity (LC) of doxorubicin-loaded mixed micelles (polymer concentration was 6 mg/mL).

Doxorubicin (mg/mL DMSO)	Particle size (nm)	PDI	Drug content (%)	Loading efficiency (%)
1	318.5±13.6	0.582±0.063	1.5	10.8
2	225.3±1.2	0.339±0.017	5.6	22.5
3	165.2±6.3	0.209±0.063	12.9	38.7
4	289.8±4.9	0.291±0.018	18.4	45.8
5	306.4±9.7	0.361±0.028	20.6	45.4
6	318.8±19.8	0.556±0.089	23.5	46.9

S-7. Drug loading and releasing profiles

In our study, the potent anticancer drug, doxorubicin (Dox), was loaded into the L4 polymeric mixed micelles. In advance, the various concentrations of doxorubicin hydrochloride were independently neutralized using a 1.2 molar excess of triethylamine in DMSO to remove the salt form. To prepare mixed micelles, a total of 6 mg of the polymers, comprising 33 wt % of graft copolymer L4 and 66 wt % of mPEG-b-PLA, was dissolved in the solutions with various drug concentrations, followed by dialysis against Milli-Q water. Afterward, the particle size and PDI of the mixed micelles were measured using DLS. The Dox-loaded polymeric mixed micelles were freeze-dried and re-dissolved in DMSO to determine the drug loading content (LC) and encapsulation efficiency (EE).

As shown in **Table S3**, the DLS measurements indicated that the particle sizes are all within the nanometer scale, precisely located in the range from 212 to 320 nm. When 3 mg/mL of Dox was fed in, the smallest particles and PDT were detected. The smallest particles of the Dox-loaded mixed micelles were 165.3 ± 6.3 nm, whereas the lowest PDI value was 0.21 ± 0.063. Additionally, the loading content (LC) and encapsulation efficiency (EE) were measured, and are reported in Table S3. The drug content and loading efficiency increased with loading Dox concentration. Doxorubicin was encapsulated in the micelles at a polymer concentration of 6 mg/mL with an efficiency of 38-46% for higher concentrations (3–6 mg/mL) of Dox infeed. However, the particle size and PDI also increased with loading Dox concentrations. Presumably, the remainder rapidly precipitated at higher concentrations. Considering the particle size and drug loading efficiency and content, at a drug loading concentration of 3 mg/mL, satisfactory Dox-loaded mixed micelles were acquired. Moreover, the morphology of the Dox-loaded mixed micelles was confirmed using TEM after negative staining. The TEM images in **Figure S7a** in the Supporting Information show that the Dox-loaded

mixed micelles have spherical core-shell structures, whose particle sizes agree with the DLS results. This finding illustrates that the loading of Dox did not disrupt the micellar structures and that Dox could be encapsulated into the mixed micelles.

The drug-releasing profiles of the Dox-loaded mixed micelles in different pH environments were further evaluated. To mimic physiological conditions, the acidic tumor microenvironment, and the endocytic process in cancer cells, the Dox-loaded mixed micelles were placed in a dialysis bag and independently incubated at pH 7.4 and 6.6. Additionally, to imitate the endocytic process in cancer cells, the Dox-loaded mixed micelles that were placed in a dialysis bag were also incubated at pH 5.4 and 4.5 for 72 h at 37 °C. The released Dox was detected using the UV–Vis spectrum and the drug-release profiles are shown in **Figure S7b**. After the initial 6 h, the drug-release profiles were similar, but after 18 h of incubation, the drug release was dependent on the environmental pH. At 18 h post-incubation, the Dox-loaded mixed micelles released two-fold higher levels of Dox in pH 4.5 in comparison to those incubated in pH 7.4. With increasing incubation duration, the Dox-loaded mixed micelles in pH 4.5 and 5.5 released more Dox, whereas the Dox-loaded mixed micelles in pH 7.4 gradually released their payloads. The rapid and pH dependent releasing profiles are mainly led from the histidine molecules in the graft copolymers. At 72 h post-incubation, the Dox-loaded mixed micelles in pH 7.4 only released 18.6% ± 1.2% of the encapsulated Dox and those in pH 6.6 released approximately 25% of the Dox, which may lead from the slow degradation of the copolymers. The Dox-loaded mixed micelles in pH 5.5 and 4.5 both released over 50% of the Dox. The Dox-releasing profiles clearly demonstrated that in mimetic physiological conditions (pH 7.4), the mixed micelles maintain stability to protect their payloads and gradually release their payloads in a mimetic tumor microenvironment, while the polymeric mixed micelles release a considerable amount

of Dox in mimetic intracellular conditions during endocytosis. The Dox-loaded mixed micelles possess the ability to release drugs intracellularly.

(a)

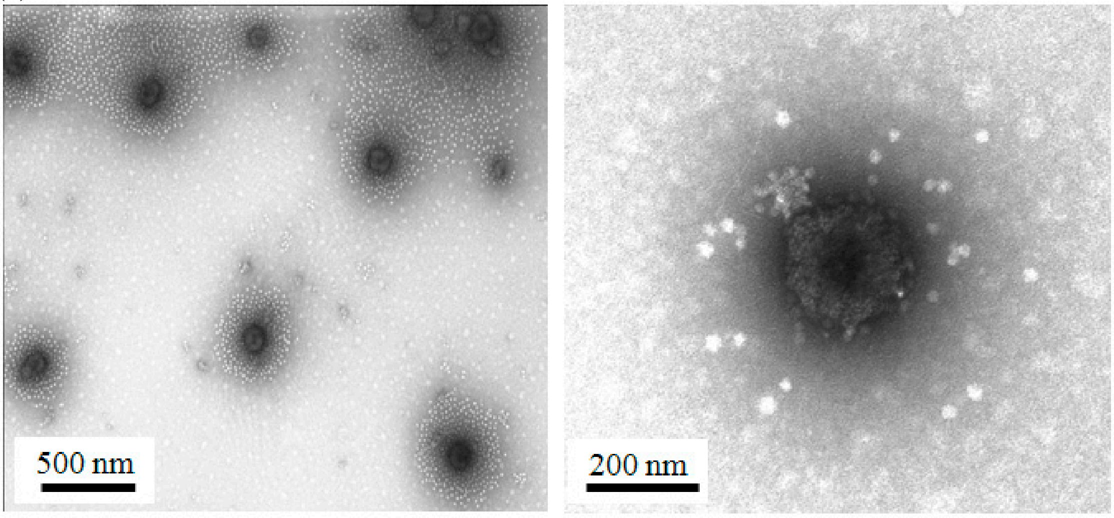

(b)

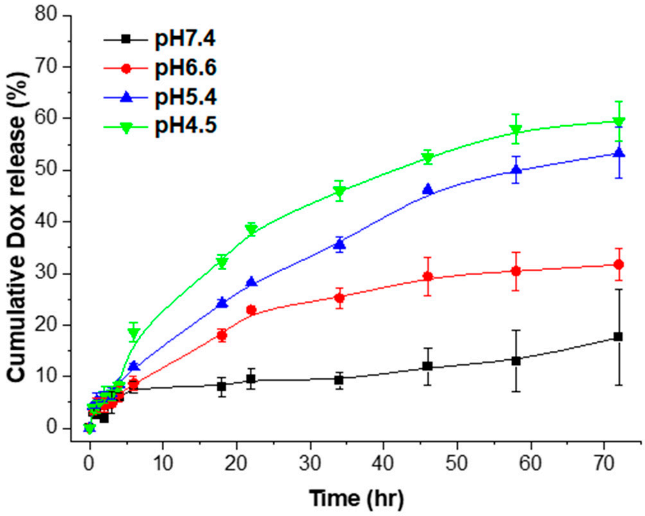

Figure S7. The morphology and drug-releasing profile of the Dox-loaded mixed micelles. (a) TEM images of the Dox-loaded mixed micelles. (b) The drug-releasing profiles of the Dox-loaded mixed micelles in different pH conditions (pH 7.4, 6.6, 5.4, and 4.5). The results are presented as mean ± SD (n = 3).

Table S4. IC_{50} values for free doxorubicin, G4, and G9 Dox-loaded micelles for cancer cells.

Cell line	IC_{50} (μg/mL)			
	Doxorubicin		Dox-loaded mixed micelles	
	24 h	72 h	24 h	72 h
ES2	0.92	31.2	0.32	8.9
LL/2	0.051	0.88	0.89	0.89
